# Rare Case of Additional Ileocecal Resection for Ascending Colon Cancer with R1 Resection due to Advanced Perineural Invasion

**DOI:** 10.70352/scrj.cr.25-0016

**Published:** 2025-05-08

**Authors:** Yoshiaki Kanemoto, Tomonari Amano, Tomohiro Kurokawa, Tetsuya Tanimoto, Masahiro Amano, Kunihisa Miyazaki

**Affiliations:** 1 Department of Surgery, Jyoban Hospital of Tokiwa Foundation, Iwaki, Fukushima, Japan; 2 Department of Pathology, Tokyo-Kita Medical Center, Tokyo, Japan; 3 Navitas Clinic, Tokyo, Japan; 4 Department of Surgery, Tokyo-Kita Medical Center, Tokyo, Japan

**Keywords:** ascending colon cancer, perineural invasion, lateral progression, R1 resection, additional resection, adjuvant chemotherapy

## Abstract

**INTRODUCTION:**

Perineural invasion (PNI) has been cited as an independent prognostic factor in colorectal cancer. We report the first case of an additional resection after ileocecal resection due to advanced lateral extension of PNI, with a review of the literature.

**CASE PRESENTATION:**

A 67-year-old woman underwent colonoscopy due to positive fecal occult blood. Biopsy revealed a 20-mm type 2 tumor in the ascending colon near the ileocecal valve, which was a poorly differentiated adenocarcinoma. She underwent laparoscopic-assisted ileocecal resection and D3 dissection, and the surgery was completed routinely in which functional end-to-end anastomosis (FEEA) was performed extracorporeally. Postoperative course was good and she was discharged one week postoperatively. The pathology showed AI, type 3, 30 × 23 mm, 40%, por2>sig>tub2, pT3a (SS), int, INFb, v2, ly3, Pn1b, PM1, DM0, pN1. There was widespread cancerous extension along the intermuscular plexus within the intrinsic muscular layer of the ileum, and although grossly separated from the tumor by about 80 mm, the tumor was R1 resected with positive oral margins. Additional anastomotic resection was performed by laparotomy. Intraoperatively, the resected section was submitted to a rapid examination, which was confirmed to be negative, and the surgery was completed. The pathological examination revealed that the resected specimen showed an adenocarcinoma on the ileum side of the anastomosis, which infiltrated and proliferated within the intermuscular plexus by about 15 mm, although the tumor was not visually recognized on the resection specimen. Both bilateral margins were negative, resulting in R0 resection. Postoperative adjuvant chemotherapy was not requested by the patient. Thereafter, periodic imaging follow-up was performed and, nine months after the initial diagnosis, there was no increase in tumor markers and no evidence of recurrence on imaging.

**CONCLUSIONS:**

Preventing R1 resection due to lateral extension of advanced PNI, which is very rare as in this case, is practically difficult given its frequency and residual bowel function. Instead, prompt additional resection and adjuvant therapy (which was not performed in this case) are essential to minimize the risk of recurrence.

## Abbreviations

CI
: confidence interval
DFS
: disease-free survival
FEEA
: functional end-to-end anastomosis
LRM
: longitudinal resection margin
NAC
: neoadjuvant chemotherapy
NCAM
: neural cell adhesion molecule
RR
: relative risk
OS
: overall survival
PNI
: perineural invasion
PS
: performance status

## INTRODUCTION

There are several routes that allow the spread of tumor cells, including direct growth, dissemination through the blood and lymph nodes, and growing along the nerves. The nerve invasion is called PNI, which is a route for metastatic spread in various cancer types, including colorectal cancer.^1)^ It is listed as an independent prognostic factor among the pathological stages of colorectal cancer in the National Comprehensive Cancer Network (NCCN)^2)^ guidelines. Indeed, as the guideline cites, a meta-analysis that included 58 studies and 22900 patients showed that PNI is correlated with a worse 5-year OS (RR, 2.09; 95% CI, 1.68–2.61) and 5-year DFS (RR, 2.35; 95% CI, 1.66–3.31).^3)^ In the present case, a routine ileocecal resection for ascending colon carcinoma was performed, and pathological findings revealed PNI. In addition, we have experienced the case in which a lateral extension along the plexus in the intrinsic muscularis propria of the ileum resulted in a positive oral margin and R1 resection, despite the fact that the resection was performed at a sufficient distance (80 mm from the tumor). This has not been reported previously, and we report the case with a literature review.

## CASE PRESENTATION

A 67-year-old, female, no specific chief complaint.

History: gastric cancer (pyloric gastrectomy and B-I reconstruction at age 40), hiatal hernia of esophagus, tinea cruris.

Current medical history: Due to the positive result of occult blood in stool at physical checkup, she underwent colonoscopy at a local doctor 28 days prior to her visit to our hospital. A 20-mm size type 2 tumor was found in the ascending colon near the ileocecal valve, and biopsy revealed a poorly differentiated adenocarcinoma, so she was referred to our hospital for surgery.

Findings on admission: height 145.8 cm, weight 38.6 kg, performance status: PS 0.

**Fig. 1:**
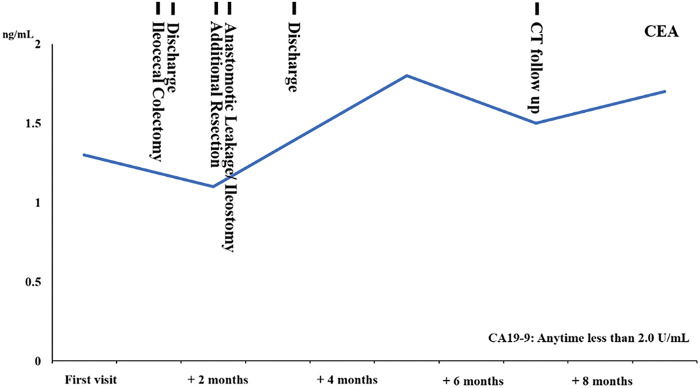
The table shows CEA trends and clinical events from the time of the first visit.

Laboratory findings: Hb 12.7 g/dL and no significant anemia. No hepatic dysfunction, no renal dysfunction. No elevation of tumor markers: carcinoembryonic antigen (CEA): 1.3 ng/mL, CA19-9: 2.0 U/mL (**Fig. 1**).

**Fig. 2:**
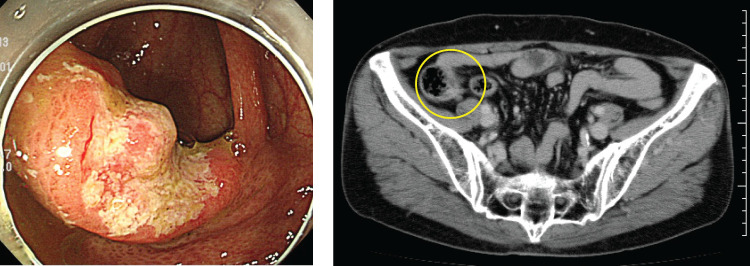
Left panel: Colonoscopy images are shown. Right panel: One slice of contrast CT images is shown. The yellow circle indicates the lesion.

Contrast-enhanced computed tomography scan: Early thickening of the wall in the ileocecal area. Depth of disease suspicious for cT3(SS). No significant lymph node enlargement. No liver or lung metastases noted. No pleural effusion, no ascites (**Fig. 2**).

Colonoscopic findings: A 20-mm area of erosion with a nodular elevation at the Bauhin’s valve (type 2) was observed. The entrance to the Bauhin’s valve could not be identified. Biopsy revealed a poorly differentiated adenocarcinoma (**Fig. 2**). Positron emission tomography/computed tomography was not performed preoperatively. A barium enema examination was not performed.

Preoperative diagnosis: cT3N0M0, stage IIA (NCCN guidelines Ver 5.2024).

**Fig. 3:**
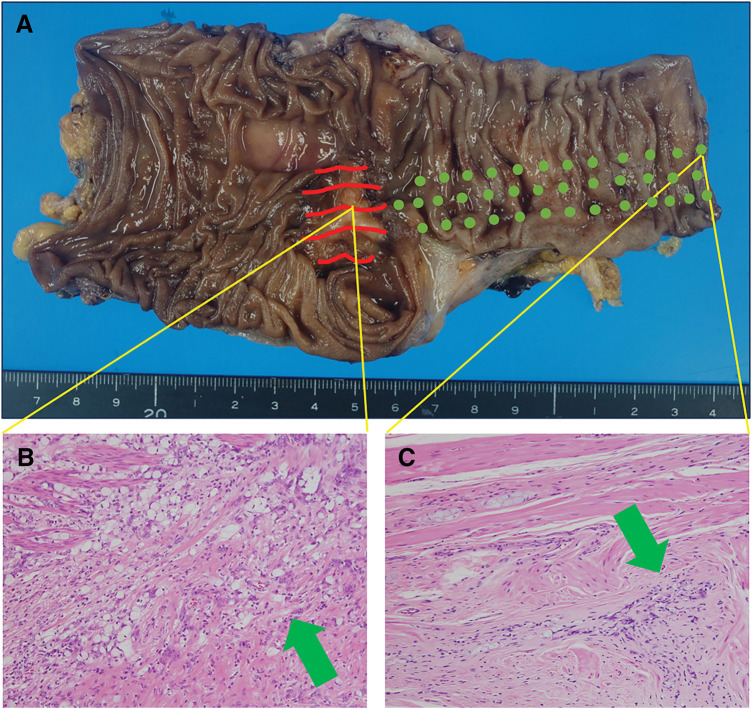
(**A**) Resection specimen. The red lines show the lesion, and the green dots indicate progress in peripheral nerve invasion; (**B**) Center part of tumor. There is an invasive growth of poorly differentiated adenocarcinoma mixed with signet ring cell carcinoma; (**C**) Near the specimen margin, with nerve invasion and intermuscular invasion of carcinoma similar to that of the main lesion.

**Fig. 4:**
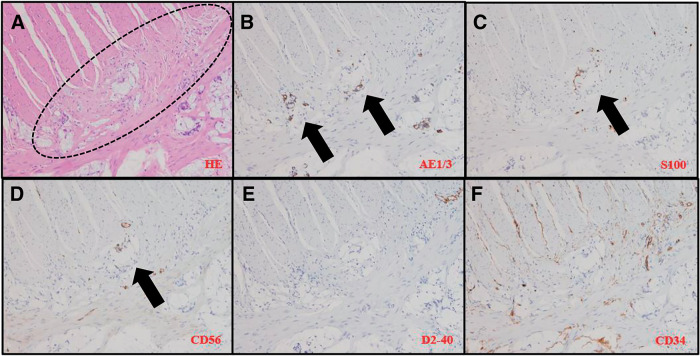
(**A**) Muscular plexus, inside dotted line; (**B**) Cancer cells identified; (**C**) Schwann cells identified. Cancer cells are distributed in many areas other than the S100-positive area; (**D**) Neural cell adhesion molecule (NCAM) is almost absent in cancer cells; (**E, F**) No vascular or lymphatic invasion, no extensive extension due to vascular invasion.

Progress: 13 days after the initial visit, a laparoscopic-assisted ileocecal resection was performed. The patient had a good postoperative course and was discharged 1 week postoperatively. Surgical findings were D3 dissection, with 80-mm oral and 100-mm anorectal resection from the lesion. The appearance of the intestine and mesentery was normal. The tumor was a 30 × 23 mm type 3 lesion extending from the Bauhin’s valve toward the anal side, with a distance of 0 mm from the Bauhin’s valve. A total of 23 lymph nodes were dissected, and metastasis was identified in 3 nodes. Histopathology findings were AI, type 3, 30 × 23 mm, 40%, por2>sig>tub2, pT3a(SS), int, INFb, v2, ly3, Pn1b, PM1, DM0, pN1, widespread cancerous extension along the intermuscular plexus within the ileal intrinsic muscle layer with positive oral margins about 80 mm from the tumor grossly, resulting in R1(fStage: IIIB, NCCN guidelines Ver 5.2024). No difference was detected between the specimen and the surrounding normal tissue on palpation as well as visual examination (**Fig. 3**). Pathological results showed cancer cells were present in the S100-positive area, but not in the S100-negative area. There were areas where cancer cells are seen intermingled with Schwann cells, confirming cancerous extension along the intermuscular plexus (**Fig. 4**). By definition, it can be a PNI.^3)^ Forty-five days after the initial visit, an additional anastomotic resection was performed by laparotomy, and the abdominal cavity was highly adherent. The resection was 50 cm on the oral side and 20 cm on the anorectal side, which was submitted to rapid pathology intraoperatively. Enteroscopy was not performed. And again, functional end-to-end anastomosis (FEEA) was performed after the resection having been confirmed negative. Histopathological findings revealed adenocarcinoma infiltrating and growing within the intermuscular plexus about 15 mm on the ileal side of the anastomosis, although the tumor was not recognized grossly on the resection specimen (no figure). Both bilateral margins of the resection specimen were negative. Anastomotic leakage occurred after the second additional resection and re-anastomosis surgery. During this procedure, an inflammatory nodule was observed at the root of the mesentery, and the terminal ileum showed poor coloration, suggesting impaired blood flow. An indocyanine green (ICG) blood flow evaluation was not performed. Anastomotic resection and ileostomy were performed on the 10th postoperative day. After another 40 days, the patient was discharged from the hospital. Postoperative adjuvant chemotherapy was not requested by the patient. Blood samples and imaging follow-up were performed regularly, and even now, 9 months after the initial diagnosis, tumor markers have not shown any abnormal values, and there has been no evidence of recurrence on imaging. A laparotomy is scheduled to be performed approximately 1 year after the surgery. At that time, anastomosis will be performed by FEEA after confirming the absence of gross abnormalities.

## DISCUSSION

In the present case, we performed a routine ileocecal resection for ascending colon cancer, but additional resection was required because lateral extension of the cancer was observed along the intermuscular plexus within the intrinsic muscular layer of the ileum (not at all discernible from the appearance), and a positive oral margin resulting in R1 were observed despite a grossly visual resection at a sufficient distance of about 80 mm from the tumor.

Originally, PNI was known as a pathway of cancer progression in head and neck tumors, pancreatic cancer, and prostate cancer, and was reported to cause shorter survival and higher local recurrence in head and neck tumors.^4,5)^ In colorectal cancer, it was first reported by Seefeld et al. in 1943,^6)^ and Ueno et al.^7)^ described PNI as cancer spread along the nerves of the Auerbach's plexus as an intramural perineural invasion, and extramural perineural invasion as tumor cell invading or spreading along the nerve fascicles external to the muscularis propria. The mechanism by which this occurs is generally understood to be that cancer cell growth is stimulated by receptors for autonomic neurotransmitters and is mediated by activation of the corresponding signaling pathways. However, the mechanism has not been fully elucidated in colorectal cancer, and the abundance of autonomic nerve fibers may be involved.^8)^

PNI is currently listed as an independent prognostic factor among the pathological stages of colorectal cancer in the NCCN,^2)^ and its presence is associated with a higher risk of recurrence and worse prognosis. Indeed, Knjin N et al.^3)^ described in a meta-analysis that included all stages in 58 studies and 22,900 patients that PNI was associated with a worse 5-year OS (RR, 2.09; 95% CI, 1.68–2.61) and 5-year DFS (RR, 2.35; 95% CI, 1.66–3.31).

Although PNI was not diagnosed on preoperative biopsy in the present case, there are no data to recommend neoadjuvant chemotherapy: NAC in resectable colon cancer patients with PNI on biopsy. There are no reports of preoperative diagnosis of advanced PNI with lateral extension as in the present case. However, there are reports that in Bulk nodal or T4 cases, neoadjuvant chemotherapy with 3 cycles of FOLFOX or CAPEOX resulted in a significant reduction in progression (*P* = 0.04) and acceptable toxicity.^9–11)^ In any case, the NAC recommended conditions were not met in this case.

Next, we discuss the extent of resection. In the present case, resection was performed at a distance of 80 mm from the oral and 100 mm from the anorectal segments. S Y Lee et al.^11)^ investigated the extent of resection in 1343 primary colon cancer patients without distant metastasis who underwent curative resection. Patients were classified into three groups: LRM <3 cm (n = 186), LRM ≥3 and <5 cm (n = 376) and LRM ≥5 cm (n = 781) and the 3-year DFS (89.2%, 89.0%, and 87.0%; *P* = 0.629) and 5-year OS (89.0%, 92.1% and 91.8%; *P* = 0.679) did not reach statistical significance. Therefore, it can be said that the extent of the resection was reasonable in this case.

Regarding lymph node dissection, it is known from population-based studies^12,13)^ that the goal is to evaluate 12 or more lymph nodes, which has been shown to improve survival. In the present case, D3 dissection was performed, and the number of dissected lymph nodes was reasonable.

Finally, postoperative adjuvant chemotherapy is discussed. The patient had SS depth of tumor, poorly differentiated adenocarcinoma histology, and PNI, and was at high risk of recurrent metastasis. This case is stageIIIB with pT3N1M0, and adjuvant chemotherapy is recommended.^2)^ However, at the patient's request, blood samples and imaging follow-up were performed without adjuvant therapy, and even now, 9 months after the initial diagnosis, tumor markers have not shown any abnormal values and there is no evidence of recurrence on imaging.

We conducted a literature search on PubMed using the keywords “perineural invasion” and “additional resection”, which yielded eight results. However, none of these reports described a case similar to ours. Notably, one report^14)^ described a case in which an appendectomy for appendicitis revealed goblet cell adenocarcinoma with negative surgical margins. Upon additional ileocecal resection, PNI was detected as a skip lesion in the cecum. To further investigate the pathological findings of our case, we expanded our search using the keywords “perineural invasion” and “poorly differentiated adenocarcinoma”, which yielded 20 results. Similarly, searching for “perineural invasion” and “signet-ring cell carcinoma” returned 50 results. However, we did not find any studies specifically analyzing the relationship between these histological subtypes and PNI. To establish evidence that may help prevent the need for additional resection in such cases, further analyses involving a larger number of cases are required.

In light of the above, it is difficult to prevent R1 resection due to lateral extension of PNI that is not at all discernible from the appearance, which is very rare as in this case, considering the frequency of such cases, preoperative examination methods, and residual bowel function. If this is found to be the case, efforts should be made to minimize the risk of recurrence by performing additional resection and adjuvant therapy as soon as possible, considering the high risk of recurrence.

## CONCLUSIONS

We presented the case report of a patient who underwent a routine ileocecal resection for ascending colon cancer, but required additional resection due to a positive resection margin caused by advanced PNI along the intermuscular plexus within the intrinsic muscular layer and its lateral extension. Prompt additional resection and postoperative chemotherapy are crucial in cases of R1 resection. This is particularly important for patients with PNI, as they are at high risk of recurrence and have a poor prognosis. Furthermore, detecting lateral extension through preoperative biopsy remains a significant challenge.

## DECLARATIONS

### Funding

None.

### Authors’ contribution

Conceptualization: YK.

Data curation: YK, TA, and TK.

Writing—original draft preparation: YK.

Writing—review and editing: YK, TA, TK, TT, MA, and KM.

Visualization: YK, TA, TK, and MA.

Supervision: MA and KM.

All authors have read and agreed to the published version of the manuscript.

### Availability of data and materials

Not applicable.

### Ethics approval and consent to participate

This work does not require ethical considerations or approval. Informed consent to participate in this study was obtained from the patient.

### Consent for publication

Written informed consent was obtained from the patient for the publication of this case report and accompanying images.

### Declaration of competing interest

Some authors have disclosed the following potential conflicts of interest: TT has received advisory fees/rewards from Medical Network Systems Inc. KM holds a leadership position in Tokyo-Kita Medical Center, which could be considered a potential conflict of interest related to the findings of this study. The other authors declare no competing interests.
